# Raynaud’s Phenomenon: Reviewing the Pathophysiology and Management Strategies

**DOI:** 10.7759/cureus.21681

**Published:** 2022-01-28

**Authors:** Iqra Nawaz, Yashfa Nawaz, Eisha Nawaz, Muhammad Romail Manan, Adil Mahmood

**Affiliations:** 1 Medicine, Quaid-e-Azam Medical College, Bahawalpur, PAK; 2 Medicine, Army Medical College, Rawalpindi, PAK; 3 Medicine, Services Institute of Medical Sciences, Lahore, PAK; 4 Medicine, Bahawal Victoria Hospital, Bahawalpur, PAK

**Keywords:** rheumatology, pathology, cold fingers, hereditary raynaud phenomenon, raynaud disease

## Abstract

Raynaud’s phenomenon (RP) is a multifactorial vasospastic disorder characterized by a transient, recurrent, and reversible constriction of peripheral blood vessels. RP is documented to affect up to 5% of the general population, but variation in its prevalence is commonly recognized owing to many factors, including varied definitions, gender, genetics, hormones, and region. Furthermore, RP may be idiopathic or be a clinical manifestation of an underlying illness. Patients with RP classically describe a triphasic discoloration of the affected area, beginning with pallor, followed by cyanosis, and finally ending with erythema. This change in color spares the thumb and is often associated with pain. Each attack may persist from several minutes to hours.

Moreover, the transient cessation of blood flow in RP is postulated to be mediated by neural and vascular mechanisms. Both structural and functional alterations observed in the blood vessels contribute to the vascular abnormalities documented in RP. However, functional impairment serves as a primary contributor to the pathophysiology of primary Raynaud’s. Substances like endothelin-1, angiotensin, and angiopoietin-2 play a significant role in the vessel-mediated pathophysiology of RP. The role of nitric oxide in the development of this phenomenon is still complex. Neural abnormalities resulting in RP are recognized as either being concerned with central mechanisms or peripheral mechanisms. CNS involvement in RP may be suggested by the fact that emotional distress and low temperature serve as major triggers for an attack, but recent observations have highlighted the importance of locally produced factors in this regard as well. Impaired vasodilation, increased vasoconstriction, and several intravascular abnormalities have been documented as potential contributors to the development of this disorder. RP has also been observed to occur as a side effect of various drugs.

Recent advances in understanding the mechanism of RP have yielded better pharmacological therapies. However, general lifestyle modifications along with other nonpharmacological interventions remain first-line in the management of these patients. Calcium channel blockers, alpha-1 adrenoreceptor antagonists, angiotensin-converting enzyme inhibitors, nitric oxide, prostaglandin analogs, and phosphodiesterase inhibitors are some of the common classes of drugs that have been found to be therapeutically significant in the management of RP. Additionally, anxiety management, measures to avoid colder temperatures, and smoking cessation, along with other simple modifications, have proven to be effective non-drug strategies in patients experiencing milder symptoms.

## Introduction and background

Raynaud’s phenomenon (RP), a commonly prevalent but often unrecognized vasospastic disorder, was discovered by Maurice Raynaud in 1862, who explained this phenomenon as part of his doctoral thesis, in a female patient presenting with transient digital ischemia [1]. The patient felt her fingers become senseless and acquired a whitish-yellowish hue on exposure to the cold. These vascular events ended with a painful experience, ultimately returning to normal after some time [1,2]. Thus, the initial description of RP given by the French doctor who described 25 cases of increasing severity, was that it is a recurring and symmetrical ischemic attack precipitated by low temperatures and/or certain emotional triggers [3]. These vascular events were then labeled as “Raynaud’s disease (RD)” in 1883 [3]. Later on, as multiple etiological factors were found to be contributing to the vasoconstriction observed in this condition, this disease was appropriately referred to as “Raynaud’s phenomenon” [3]. In view of recent research, RP is recognized as a transitory, episodic, and reversible vasospasm of peripheral blood vessels [4], characterized by color changes ranging from white caused by constriction of digital arteries (ischemic phase) to bluish-cyanotic (cyanotic phase) and ultimately to red due to vasodilation (hyperemic phase) [5]. RP can be an exacerbated physiological response to a cold, or a clinical sign or symptom of an underlying secondary illness, or it may simply be a serious sign of severe ischemia of a digit. Hence, a clear distinction must be established between RP and other vasospastic disorders such as acrocyanosis, in which cyanosis is also precipitated by lower temperatures [6]. For building a gold-standard treatment approach, it is imperative to have a thorough understanding of the pathophysiology of this phenomenon, for which several vascular and neural abnormalities have been elucidated, along with associated factors that tend to precipitate these vasospastic episodes. Therefore, this review aims to present the existing literature on Raynaud’s phenomenon, using Fredrick Wigley’s works [1] as a foundation, and offer relevant insight on the recent data from the last few years.

## Review

Prevalence

According to recent statistics, it is widely believed that 3-5% of the general population is affected by RP, despite a great degree of variation in prevalence [7]. This variation is based on the differences in how this phenomenon is defined. These differences in definition ultimately influence the diagnosis. Moreover, as these vasospastic episodes rarely take place in the presence of a physician, statistics are heavily based on the history provided by the subjects [8]. Epidemiology is also affected by climatic and geographical factors [7,9], with colder regions being associated with a higher rate of prevalence [10]. A pattern of familial predisposition to RP has also been observed, as in some cases, patients do recount a relative with the same set of symptoms [11]. RP has been recognized more widely in women as compared to men, thus denoting a gender bias [12]. The onset of RP is observed at a relatively younger age in women, thus indicating that young females are more likely to be at risk [12]. Furthermore, RP is observed in about 95% of patients suffering from systemic sclerosis (SSc) or scleroderma and is one of the first clinical manifestations and often a heralding symptom of this disease [13].

Aggravating factors

Enhanced susceptibility to cold markedly increases the incidence of RP as low temperature triggers peripheral vasoconstriction leading to transient ischemia of the exposed digits [14]. Vasospasm sets in when the temperature falls below a certain cutoff temperature. This cutoff temperature is specific for each patient [9]. A low body mass index also increases the prevalence of RP as it increases the patient’s sensitivity to cold [12,15]. Besides cold, emotional stress is another strong trigger that can elicit vasospastic episodes. In a study carried out to investigate the contribution of cold and stress in triggering RP, it was observed that one-third of these attacks were aggravated by stress [16]. This finding is in confirmation of the observation that emotional upset and stress can also cause tachycardia and digital vasoconstriction [17,18], thus adding to the probability of a vasospastic episode.

Hormones, mainly estrogen, are believed to be associated with a higher frequency of vascular disorders as they potentiate the vasoconstriction brought about by the alpha adrenoreceptors [12]. A high prevalence of RP has been demonstrated in premenopausal females [19], owing to the observation that estrogen increases the expression of α2C-adrenoreceptors by activation of α2C-adrenoreceptor gene promoter [20]. Moreover, unopposed estrogen therapy increases the risk of the incidence of RP in postmenopausal women [21]. RP has also been demonstrated to be genetically predisposed among families; this is given by the observation that whole genome screening of six extended families with multiple RP patients revealed five chromosomal regions of possible linkage found to be associated with the predisposition of RP [22]. In addition to these factors, occupational exposure to vibratory tools is also deeply linked with an elevated risk of RP, which is why RP may also be termed as “hand-arm vibration syndrome” or “white finger syndrome” [12,23]. Recently, reports of RP prevalent in guitarists and a slap bass player have also been presented [24]. Furthermore, chemicals such as vinyl chloride monomer (VCM) [25], arsenic [19], and nicotine [26] have also been observed to be associated with RP.

Smoking and age have been reported to affect the incidence of RP in men only. This is in contrast to marital status and alcohol intake being the major risk factors in women only [27]. Divorce, separation, and the death of a spouse add to emotional stress and thus can aggravate RP [28]. Alcohol has been hypothesized to increase estrogen levels and hence partakes in augmenting Raynaud’s attack, particularly in women [12,27].

Phases of Raynaud’s phenomenon

The three stages of RP that have been described include ischemia owing to the vasoconstriction of the affected area, hyperemia due to the recovery of compromised blood perfusion, and finally a normal state of blood flow [9]. Thus, we observe a white phase due to cessation of blood supply, a bluish cyanotic phase due to desaturation of residual blood, and finally, a red phase owing to the restoration of reduced blood flow [29,30]. The white or blue attack persists for about 20 minutes [31]. This triphasic change, however, is not always observed. In accordance with the guidelines issued by the European Society of Vascular Medicine in 2017, whitish discoloration (blanching) must be present in order to establish a diagnosis of RP, as cyanosis and rubor may or may not be present [14]. However, some authors tend to consider the presence of cyanosis as a sufficient criterium for diagnosis [32].

Classification of Raynaud’s phenomenon

Several approaches have been introduced to classify RP. The current approach aims to classify it into primary RP or idiopathic RP (also known as Raynaud’s disease (RD)), and secondary RP (also known as Raynaud’s syndrome (RS)) [8]. Secondary RP is often a clinical manifestation of an underlying disease. Contrary to this, if no underlying illness is detected, then it is labeled as primary RP [8]. Thus, this approach to classification categorizes all disease conditions associated with peripheral vascular compromise under the heading of "secondary RP" and labels the remaining patients with primary Raynaud’s disease [33].

Mechanism of Raynaud’s disease

Since RP involves a transient compromise of blood flow to the affected area, it has been believed to be mediated by either neural or vascular mechanisms. A neural mechanism that has been postulated for RD includes an increased activity of the sympathetic nervous system upon stimulation by cold or stress. Norepinephrine is released and causes the activation of postjunctional alpha adrenoreceptors located on the vascular smooth muscle that mediate vasoconstriction [29]. This is in alliance with the mechanism proposed by Raynaud that ischemic episodes are precipitated by overactivity of the noradrenergic nerves in the skin, as sympathetic stimulation is increased on exposure to cold and under emotional stress. However, neural mechanisms are overridden by local factors. Furthermore, Raynaud’s attack observed in patients after local anesthesia of digital sympathetic nerves or following sympathectomy is suggestive of the fact that there is a "local fault" in digital vessels that also contributes to the transient peripheral vasospasm [29,34].

Cold-induced vasoconstriction is propagated by the production of reactive oxygen species (ROS) by the mitochondria. This organelle senses the temperature and serves as a thermo-sensor in the vascular smooth muscles. Upon stimulation by low temperatures, it causes release of ROS, triggering a redox signal, and stimulating the Rho/Rho-kinase pathway, which causes translocation and activation of α2C-adrenoreceptors with subsequent vasoconstriction [35,36].

Pathophysiology

Both neural and vascular effects are deemed to play a part in establishing the pathophysiology of this condition. As RP can be idiopathic or due to a secondary cause, the pathophysiology in both cases somewhat varies. Primary RP has been recognized to be related to functional alterations and is recognized as an isolated vasospastic condition, while secondary RP associated with a systemic disease has several factors contributing to its etiology. Some of these include endothelial cell injury, a disparity in the production of vasoactive substances, structural abnormalities, intravascular lesions causing cessation of blood flow, and increased vasoconstriction [37,38]. It is convenient to describe the pathophysiology under the following headings [34].

Vascular Abnormalities 

Vascular abnormalities can be seen in two aspects: structural alterations and functional alterations. Structural changes in the microvasculature and digital arteries are recognized more frequently in secondary RP. Alterations in the structure, such as a severe fibrotic proliferation of the innermost layer of the arteries (intima), have been identified in the vasculature of patients with SSc-associated RP, along with a fibrous intimal lesion that can occlude the lumen [39], thus causing cessation of blood flow to the periphery. This also renders an injury to the endothelial cells, causing endothelial dysfunction, which has also been reported to be present in the skin of SSc patients [40]. These damaged endothelial cells can aggravate vasoconstriction [41]. Expression of cytokine growth factor and activation of isolated pericytes also contribute to vascular abnormalities [42]. Thus, the pathophysiology of secondary RP differs from primary RP in that both altered microvascular structure and defective vascular function add to reducing the blood flow in the former [42]. This is in contrast to Raynaud’s disease, in which the primary fault is usually only functional [29].

Functional abnormalities producing defective endothelial function largely contribute to the pathophysiology of RP as endothelium is a very sensitive layer, reacting to and producing a large variety of vasodilators, such as nitric oxide (NO) and prostacyclin [43]. A decreased level of NO has been recognized as one of the mechanisms of pathogenesis. However, as overproduction of NO has also been observed in one study in patients with SSc [44], the role of NO is thus quite complicated. An explanation of these elevated levels of NO can be given by the fact that it may be a compensation for the diseased and nondilatable vessels [42]. Moreover, decreased production or reduced efficacy of endogenous prostacyclin may also contribute to the pathogenesis of RP [43]. The role of prostacyclin in RP is further reinforced by the observation that the vasodilating properties and antiplatelet actions of prostaglandin analogs have been demonstrated to be beneficial in the treatment of RP.

The endothelium also produces vasoconstrictive substances such as endothelin-1, which is not only a potent vasoconstrictor but also has profibrotic properties [34]. Elevated levels of endothelin-1 may also play a significant role in the pathophysiology of primary RP [45]. In addition to primary RP, elevated plasma levels of this vasoconstrictor have been reported in secondary RP as well [46]. This is further reinforced by the positive results obtained with an endothelin-1 receptor antagonist, bosentan, in primary RP and SSc [47]. Along with endothelin-1, angiotensin is another potent vasoconstrictor and a profibrotic agent produced by endothelial cells. Elevated levels of angiotensin II have been observed in patients with limited cutaneous SSc [48], which is why angiotensin-converting enzyme (ACE) inhibitors find potential in the treatment of RP [42]. In addition to this, it has also been demonstrated that in the case of SSc and possibly also in primary RP, endothelial-dependent vasodilation is reduced leading to digital ischemia [34].

Angiopoietin-2 (Ang-2) destabilizes the endothelium, causing it to become more susceptible to the inflammatory effects of cytokines and can also initiate endothelial cell death [49]. Elevated levels of Ang-2 have been indicated in many vascular diseases including SSc as well [50]. It has also been demonstrated that serum quantities of Ang-2 can be a marker of microvascular damage in patients with RP who have been treated with multiwave locked system (MLS) laser therapy [51].

Neural Abnormalities

Neural abnormalities can be studied in two aspects: central mechanisms and peripheral mechanisms. CNS influence in RP is somewhat suggested by the fact that emotional distress is one of the major triggers for a Raynaud’s attack. Raynaud himself was of the view that these vasospastic episodes occur as a result of central dysfunction [42]. However, a series of experiments have revealed that patients with primary RP do not habituate to alerting responses precipitated by emotional stress as do healthy control individuals [52,53]. Thus, they exhibit a defect in the neural process of habituation [29]. This observation contradicts the proposition that simply hyperactivation of sympathetic nervous system causes vasospasm [34].

Impaired Vasodilation

Besides nervous control, vascular tone is also regulated by the release of various substances, such as calcitonin gene-regulated peptide (CGRP), a neuropeptide [54]. CGRP has strong vasodilating properties and is released from the sensory afferents supplying the arteries. CGRP causes an increased production of adenylate cyclase, thereby increasing the levels of the second messenger, cyclic adenosine monophosphate (cAMP), which brings about the relaxation of the smooth muscle cell (SMC) [54]. A decrease in the number of CGRP-secreting neurons is observed in the skin biopsy samples taken from patients with SSc and primary RD, thus suggesting a potential involvement in the pathogenesis of RP [55]. Neurokinin A and substance P have also been observed to contribute to the pathomechanism of this phenomenon as they also increase cAMP levels, causing SMC relaxation [56]. Neuropeptide Y, a strong vasoconstrictor, has also been studied with great interest as elevated levels have been demonstrated in patients with SSc-associated RP [57].

Increased Vasoconstriction

In response to low temperatures, loss of heat from the body is prevented by decreasing the flow of blood to the skin. This reflex of the sympathetic nervous system is exaggerated in RP. This vasoconstriction is carried out by α1- and α2-adrenoreceptors in response to norepinephrine. α2-adrenoreceptors are more widely distributed in the digital arteries [58] and are exclusively found in the smooth muscles of arterioles and veins [59]. Out of the three classes of α2-adrenoreceptors, α2C-adrenoreceptor class is heavily involved in the regulation of body temperature as compared to the other two (α2A and α2B) [60]. The role of this class of adrenoreceptors is further reiterated by the fact that its selective inhibition eliminates cold-mediated vasoconstriction. Low temperature brings about spatial relocation of α2c-adrenoreceptors from the Golgi apparatus to the cell surface, and in this way it mediates vasoconstriction in response to cold [61]. This translocation of α2C-adrenoreceptors is propagated by various factors, including ROS, Rho/Rho-kinase, and the actin filament of the cytoskeleton [19].

Enhanced protein tyrosine kinase activity has also been found to be associated with RP as it has been recognized to cause cold-mediated vasoconstriction in both primary and secondary RP. This is also demonstrated by the reversal of these changes observed by inhibiting tyrosine kinase. Tyrosine phosphorylation due to increased activity of protein tyrosine kinase is a good basis for explaining the mechanism of this cold-induced vasoconstriction [62,63].

Intravascular Abnormalities

Various intravascular factors have been demonstrated to be involved either directly in the pathophysiology of RP or in simply increasing the probability of a Raynaud’s attack. These include platelet activation, impaired fibrinolysis, and oxidative stress. Elevated platelet activation and aggregation are observed in SSc as well as in primary RP [64]. This is suggested by an elevation in the levels of thromboxane and β-thrombo-globulin secreted by the platelets [34]. Furthermore, increased production of thromboxane A2, which is a strong vasoconstrictor and a powerful mediator of platelet aggregation, has been observed in patients with RP [65]. These levels have also been seen to increase on cooling [66], and also with the increasing severity of disease in secondary RP [42]. Moreover, elevated gene expression for the synthesis of thromboxane synthase was recently discovered in the leucocytes of patients with SSc [67]. Along with thromboxane A2, elevated levels of serotonin released from the platelets have also been observed. However, the role of serotonin is still under investigation as various studies have suggested contradictory results [42,68]. Impaired fibrinolysis has been demonstrated in patients with SSc-associated RP. It can aggravate the deposition of fibrin, thus causing occlusion of the blood vessel lumen [64,69]. Oxidative stress-mediated by free radicals has thoroughly been studied in RP associated with SSc and has also been found in primary RP [70]. Repeated ischemic episodes followed by reperfusion facilitate the generation of free oxygen species which precipitate tissue damage through the peroxidation of the membrane lipids. This tissue injury can activate an inflammatory cascade and exacerbate local injuries [42]. The role of oxidative stress in RP is further evident by the use of antioxidants as an approach to the reversal of this phenomenon. However, the role of oxidative stress is largely dependent on the number of attacks occurring [42]. In addition to these factors, an increased viscosity of blood has also been seen in patients with RP [71].

Clinical presentation

RP is characterized by a classic pattern of change in color in the affected area, as described previously. This triphasic discoloration of the digits includes pallor, cyanosis, and erythema, owing to the reperfusion of blood [30]. All three of these color changes may or may not be present. Raynaud’s attacks are also usually associated with pain owing to the ischemia of the sensory supply. Common complaints include pins and needles sensations and sensitivity of the digit to external stimuli [29,31].

The presence of sensitivity to cold (mainly of digits), changes in color (white or blue) on exposure to low temperatures, and emotional disturbances raise the suspicion of RP [6,30]. It is frequently seen in the extremities, most commonly in the fingers and toes, but has also been recognized in the ears, nose, and areolar tissue [33]. Recent studies have shown the occurrence of RP in the tongue [72,73] as well as in the penis [74]. However, the thumb is spared in both primary and secondary RP [33]. A vasospastic episode may subside within an hour but can also last for many hours [30].

Raynaud’s phenomenon of the nipple

Lactation experts have demonstrated that RP can either be directly involved or be a contributing factor to the pain felt during breastfeeding in lactating women [75]. Severe nipple pain along with white discoloration (blanching) of the nipple due to transient ischemia, followed by redness or bluish appearance of the affected area, are a few common symptoms that have been observed in RP of the nipple. These symptoms have also been observed to be aggravated by low temperatures and have been noted to occur at times when breastfeeding was not being done [75]. A history of cold sensitivity and the occurrence of RP on other acral surfaces can help in establishing a diagnosis of RP and can differentiate it from other types of pain associated with lactation [76]. Hence, RP can be a common occurrence in nursing mothers and must be correctly and timely recognized so that an appropriate treatment strategy can be devised that can help mitigate the pain felt during breastfeeding due to RP.

Diagnosis

**Figure 1:**
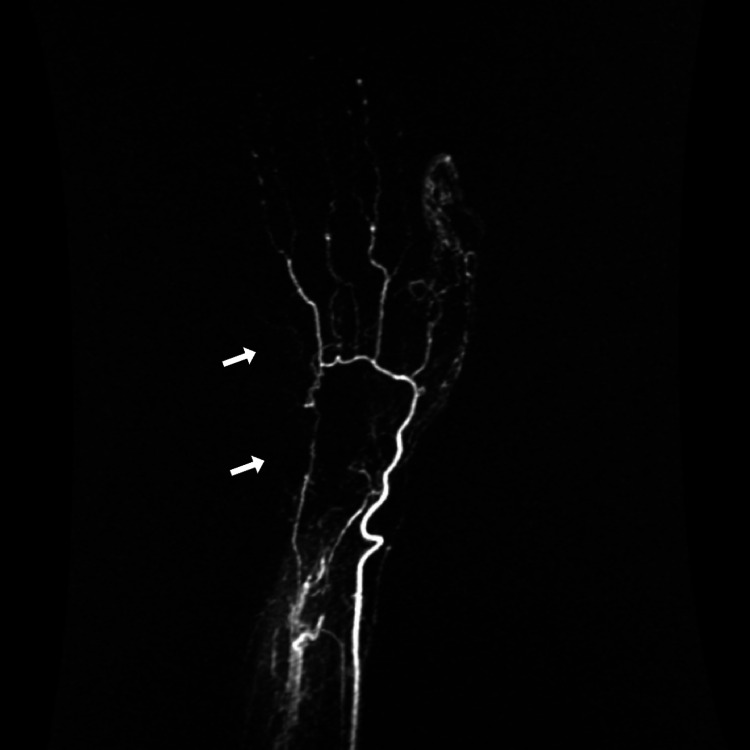
No detectable perfusion of the arteries of the fifth finger. String-of-beads appearance of the digital arteries II-IV with segmental stenoses. Narrowing of the ulnar artery in Guyon's canal. Case courtesy of Dr. Roberto Schubert, Radiopaedia.org, rID: 18201.

**Figure 2:**
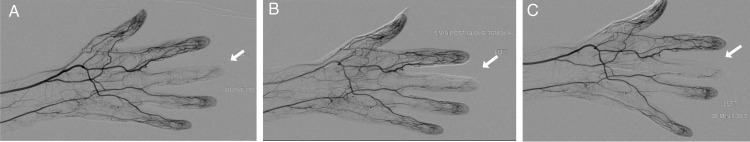
DSA hand of a 40-year-old female presents with ulceration of the distal phalanx of the middle finger. (A) Glove on; (B) five minutes post-glove removal; (C) 20 minutes post-glove removal. Arrow represents the reduced perfusion to the arteries of the middle finger. DSA: digital subtraction angiography. Case courtesy of Dr. Steven Bush, Radiopaedia.org, rID: 14327.

Diagnosis is primarily based on a detailed history given by the patient and careful observation by the examiner [77]. A history of increased sensitivity to cold and discoloration of the digits should be established. Risk factors such as age, occupation, and drug, and family history can aid in the diagnosis as well [33]. Laboratory investigations generally include a full blood count, an antinuclear antibody (ANA) test, and an erythrocyte sedimentation rate (ESR) [13]. Imaging techniques such as nailfold capillaroscopy, thermography, and laser Doppler imaging (LDI) [78] can be employed to investigate RP. In addition to these techniques, radiographic assessment of RP can be performed by Doppler ultrasound, magnetic resonance imaging (MRI) angiography, and digital subtraction angiography (DSA) [13]. Contrast-enhanced magnetic resonance (MR) angiography is a non-invasive technique that can reveal characteristic narrowing and tapering of digital blood vessels in RP [79]. However, owing to its higher spatial and temporal resolution, DSA is deemed to be the gold standard imaging technique for visualizing the microvasculature in RP [80]. Structural alterations can be observed in the magnetic resonance angiography (MRA) in Figure 1, and in DSA in Figure 2, resulting in a decreased blood flow to the digital arteries, leading to the development of RP.

Differentiating Between Primary and Secondary Raynaud’s Phenomenon

RP is not a diagnosis in itself, unless no underlying secondary cause is observed [81]. In order to establish a correct diagnosis and devise appropriate management strategies, it is extremely important to differentiate between primary and secondary RP.

The distinction between primary and secondary RP is made on clinical grounds. Symmetry observed in RP was formerly deemed as a criterium for primary RP. However, agreement on this requirement (of symmetry) was not achieved in a consensus developed recently by an international panel [82]. The panel also established that a normal ESR and a normal antinuclear antibody test were no longer mandatory for the diagnosis of primary RP [1,82]. This is in contrast to the previously published criteria, which suggested that a normal ESR and a normal ANA are required for the diagnosis of primary RP. As these tests have low specificities, the requirement for a negative ESR was completely removed and the requirement for a negative ANA was eased to either negative or low titer ANA [82]. Patients with primary RP usually do not develop complications such as digital ulcers, gangrene, or tissue necrosis. However, a loss of digital tip pulp has been reported. In the case of primary RP, no secondary cause of illness is detected.

At the nailfold, capillaries lie parallel to the skin surface and hence can be visualized for structural alterations. Nailfold capillaroscopy can be used to non-invasively visualize abnormalities in capillary morphology, number, distribution, and orientation. As primary RP is considered to be a purely functional disorder, a normal nailfold capillaroscopic examination is observed. This is in contrast to secondary RP, in which changes in capillary morphology and other structural abnormalities are exclusively reported [83]. Various approaches have been introduced for nailfold capillaroscopy, ranging from low magnification (dermatoscopy, stereomicroscopy, and USB microscopy) to high magnification (videocapillaroscopy) techniques, with each having its own advantages and shortcomings [43].

Nailfold videocapillaroscopy (NVC) is considered the gold standard approach, providing a detailed view of capillary structure. A normal shape of a capillary in a healthy individual is defined as being hairpin or tortuous. Any other shape observed on NVC is deemed abnormal hence pointing toward secondary RP [84]. In view of recent literature published regarding NVC, the presence of dilated "giant" capillaries with an apical diameter ≥ 50 μm, abnormal architecture, and an extremely low number of capillaries point to a "scleroderma pattern" of NVC, strongly suggestive of secondary RP [85,86]. A fast track algorithm has been established to differentiate between a "scleroderma pattern" and a "non-scleroderma pattern" [87]. This algorithm includes three rules that help to evaluate a capillaroscopic image [87]. Presence of giant capillaries or an extremely low capillary density (≤3 capillaries in 1 linear mm) and an abnormal capillary shape indicate a late scleroderma pattern suggestive of secondary RP [87]. The microvascular structural alterations can be categorized as "early," "active," and "late" changes. Early changes include a few dilated capillaries and microhemorrhages. Active changes include an increased number of giant capillaries and a mild loss and disorganization of capillaries. A severe loss of normal capillary structure, areas of avascularity, and abnormal neovascularization indicate the late phase of the SSc pattern [13]. Thus, capillary density, capillary dimensions, capillary morphology, and the presence or absence of hemorrhages should be observed when assessing a capillaroscopic image. These structural alterations, usually observed in secondary RP, make NVC a reliable screening tool for differentiating between primary and secondary Raynaud’s.

Secondary RP is characterized by the prevalence of painful vasospastic episodes, usually in the thirties and onwards. Clinical findings that signal an underlying hematopoietic or connective tissue disease further point toward secondary RP. Esophageal dysfunction is also common in SSc, thus suggesting secondary RP [88]. Serum analysis for autoreactive antibodies, specifically ANA, anti-centromere, and anti-RNA polymerase, indicates secondary RP. In secondary RP, cutaneous lesions including fibrosis, calcinosis, and ulcerations are usually seen [82]. Moreover, a greater risk of an underlying illness or systemic disease and a higher risk of digital loss are observed in secondary RP. Thus, predicting the risk of the occurrence of these complications can be another approach to distinguishing primary RP from secondary RP. Hence, it is imperative to establish a clear distinction between idiopathic RP and disease-associated RP so that appropriate treatment and management can be ensured.

Conditions associated with secondary Raynaud’s phenomenon and differential diagnoses

Secondary RP is characterized by an underlying illness or an associated pathology. This is reinforced by the observation that RP has been demonstrated to be a clinical sign and a presenting feature of an associated systemic disease in more than 50% of patients [33]. A high prevalence of RP has been indicated in autoimmune connective tissue diseases such as SSc or scleroderma, systemic lupus erythematosus (SLE), Sjogren’s syndrome, and mixed connective tissue disease (MCTD) [89]. RP has also been observed in patients with rheumatoid arthritis [90,91].

Secondary RP has been implicated in over 95% of patients with SSc, indicating that almost every SSc patient suffers from a typical Raynaud’s attack [92]. The microvascular damage and exaggerated vasospastic tendency in SSc are clinically manifested as RP, making RP an early symptom of this disease [93]. Besides RP, vascular injury in SSc manifests as scleroderma renal crisis and pulmonary arterial hypertension, indicating compromised blood flow to these primary organs [94]. Besides kidney and lung involvement, myocardial fibrosis is also largely observed in SSc [95] RP is also a nonspecific cutaneous manifestation in about 18-46% of patients of SLE and may precede other effects of this disease by years [96]. Thus, it facilitates the early recognition of an underlying illness, providing an opportunity for early diagnosis. RP has also been observed to occur in about 33% of patients with Sjogren’s syndrome [14], and in about 85% of patients with mixed CTD [14].

In addition to connective tissue disorders, vasospastic disorders such as acrocyanosis and perniosis with similar symptoms must also be differentiated from RP [97]. Obstructive arterial disorders, including atherosclerosis and thromboembolism, can also cause RP. Besides autoimmune diseases, conditions that increase plasma viscosity and decrease digital blood flow have also been recently reported to cause RP. These include cryoglobulinaemia, cryofibrinogenaemia, and paraproteinaemia [43]. Neurological disorders including carpal tunnel syndrome and multiple sclerosis, may also precipitate Raynaud’s attacks. Other causes may include hypothyroidism, frostbite and lesions associated with occupational use of vibratory instruments.

Drug-induced Raynaud’s phenomenon

Certain drugs have been demonstrated to cause RP as a side effect. The occurrence of RP following administration of lisdexamfetamine for treating attention-deficit/hyperactivity disorder (ADHD) has been observed recently in a 16-year-old patient [98]. Increasing the dose of methylphenidate in an adult with ADHD has also been reported to induce RP [99]. In addition to this, a recent case report has demonstrated an increased prevalence of RP as a side effect of the psychostimulant Adderall [100]. Sulfasalazine-induced RP has also been suggested as withdrawal of this drug resolves the symptoms of RP [101]. Alpha and beta interferons owing to their vasoconstrictive and procoagulant properties [102] have been demonstrated as causative agents of RP [103]. Recent studies have shown that chemotherapeutic drugs such as gemcitabine [104], vincristine, and cisplatin combined with vinblastine [19] have been reported to induce the symptoms of RP. Certain medications, including drugs for migraine, cyclosporine, and nonselective beta-blockers, can also lead to secondary RP [31]. RP of the nipple has also been reported to occur following the administration of labetalol, a first-line drug employed for gestational hypertension [105], with the symptoms resolving on discontinuation of this drug.

Management strategies for Raynaud’s phenomenon

Several treatment strategies have been devised in an attempt to develop a gold-standard approach for treating RP. This is limited by the complex pathophysiology and poorly understood etiology of this disease. Moreover, a lack of suitable animal models for the study of RP further impedes the process of experimentation and development of new drugs. Management strategies for RP depend highly on the severity of the disease. Attacks can be prevented by a non-drug approach, but pharmacologic intervention acquires importance as the disease intensifies.

Nonpharmacological Approach

As the drug-based treatment of RP is still being investigated, general and lifestyle modifications are usually the first steps in managing all patients [106]. Thus, these patients usually do not require specific therapies. Symptoms of RP can be controlled by avoiding triggers that can elicit a vasospastic attack. Avoiding the cold and keeping the body warm through thick clothing, hand warmers, and padding can prove useful. Alleviation of emotional stress and anxiety can also decrease the frequency of the onset of these attacks [97]. Cessation of smoking is also advised, as nicotine has been suggested to cause vasoconstriction [97,107].

Pharmacological Approach

Recent advances in understanding the mechanism of RP have facilitated better treatment strategies for managing the symptoms. A pharmacological approach is undertaken when nonpharmacological measures of management have been rendered ineffective in treating the symptoms associated with RP. Drug therapy can be discussed under the following headings.

Calcium channel blockers: Calcium channel blockers have long been employed in the management of RP. Of the three main classes of these drugs, which differ from one another on the basis of their cardioselectivity, the dihydropyridine group is often preferred. The dihydropyridine group, including nifedipine, felodipine, and amlodipine, is less cardioselective and hence less cardiotoxic. Nifedipine has been most thoroughly studied in this case and is often used as the drug of choice for Raynaud’s syndrome [108]. Calcium channel blockers have been demonstrated to reduce the number of vasospastic episodes by 2.8 to 5 fewer episodes per week and also the intensity of the phenomenon by 33% [109]. These drugs cause SMC relaxation by inhibition of the voltage-sensitive L-type calcium channels and thus promote peripheral vasodilation [110]. Besides calcium channel blockers, no other oral vasodilators have been proven to be efficacious in primary RP [111].

α1-adrenoreceptor antagonists: α1-adrenoreceptor antagonists such as prazosin have been demonstrated to mitigate the intensity of Raynaud’s attacks [108]. The action of norepinephrine released by sympathetic stimulation is competitively inhibited by these alpha-blockers. In a placebo-controlled study [112], it was observed that prazosin reduced the number and period of vasospastic episodes in RP and was reported to be modestly beneficial in treating scleroderma-associated RP [113].

Angiotensin-converting enzyme inhibitors: Although a clear benefit has not been reported with the use of angiotensin-converting enzyme (ACE) inhibitors [114], these drugs are still of interest as they have been demonstrated to affect endothelial function and vascular remodeling [42].

Nitric oxide: The vasodilating properties of nitric oxide (NO) hold potential for therapeutic purposes. Through cyclic-guanosine monophosphate (cGMP) modulation, NO, brings about SMC relaxation and vasodilation [42]. Several approaches have been explored, including topical application of nitroglycerin, sublingual tablets, administration of L-arginine and sodium nitroprusside, and transdermal patches. Along with some improvement in temperature and blood flow in the affected area, a high occurrence of headaches and dizziness has been observed [42,114]. Topical application of glyceryl trinitrate has been reported to decrease the number and severity of vasoconstrictive episodes in patients with primary and secondary RP [115].

Prostaglandin analogs: Prostaglandin analogs have been investigated for their potential therapeutic use in the treatment of RP due to their property of inhibiting aggregation of platelets and due to their vasodilating effects. As iloprost, an epoprostenol analog, is a strong vasodilator, with antifibrotic activity and an antiplatelet action, it has been extensively investigated for its use in severe refractory RP and in healing and preventing digital ulcerations [42]. Intravenous administration of iloprost has been reported to mitigate the intensity, number, and period of vasospastic attacks [108]. More recently, long-lasting effects of this drug have been observed, warranting a promising approach to treatment [116].

Phosphodiesterase inhibitors: As described earlier, the mechanism by which NO causes vasodilation is through the relaxation of the SMC via the action of cyclic guanosine 5′-monophosphate (cGMP). Hydrolysis of cGMP is mediated by cGMP-specific phosphodiesterase 5 (PDE5) [117]. Thus, inhibition of these phosphodiesterases can result in sustained SMC relaxation and continued cGMP-dependent vasodilation [117,118]. A few examples of cGMP-specific phosphodiesterase type five inhibitors are sildenafil, vardenafil, and tadalafil. Sildenafil has been most thoroughly investigated in its relation to RP. Various studies have revealed evidence of these PDE5 inhibitors having a potential role in treating the ischemic attacks of RP [118]. A few side effects of the use of these PDE5 inhibitors that have been observed include headaches, myalgia, rhinorrhea, visual problems, and dizziness [114].

Selective serotonin reuptake inhibitors (SSRIs): Although the contribution of serotonin in the pathogenesis has not been clearly demonstrated, alleviation of symptoms of RP has been observed with fluoxetine, possibly by blocking the uptake of serotonin into the platelets and reducing the level of serotonin released during platelet activation [119]. The severity of the attack and the frequency of the episodes were reported to be reduced with fluoxetine therapy [120] but a bilateral fixed RP has also been reported in a patient upon increasing the fluoxetine dose from her regular dose [121]. Besides fluoxetine, mitigation of symptoms has also been observed with sertraline and escitalopram [122,123]. The existing literature does suggest alleviation of symptoms with SSRIs, but citalopram-induced RP observed in a 43-year-old patient contradicts the benefits associated with these drugs [124].

Antioxidants: N-acetylcysteine, owing to its antioxidant properties, has been recognized to mitigate the severity and lessen the number of Raynaud’s attacks. The possible mechanism of N-acetylcysteine's partaking in reducing the symptoms of RP is assumed to be via modulation of adrenomedullin levels [125].

Botulinum toxin A: *Clostridium botulinum* produces botulinum toxin type A, which has been employed for relieving muscle spasms by blocking acetylcholine release. It also affects vascular smooth muscles by blocking the transmission of norepinephrine and thus inhibiting vasoconstriction. In addition to this, it blocks the recruitment of α2c-adrenoreceptors, resulting in a reduction in cold-induced vasoconstriction [126,127]. Reduced number of vasospastic attacks and increased perfusion, with interdigital injections of this toxin, has been observed in primary and secondary RP [114]. Successful treatment has also been reported with botulinum toxin B [128].

Endothelin receptor antagonist: Due to the strong vasoconstrictive properties of endothelin-1, it has been demonstrated to contribute to the pathophysiology of RP. These vasospastic effects can be inhibited by bosentan, which is a competitive antagonist of endothelin receptors. It has been observed in various studies that bosentan greatly reduces the risk of digital ulcers [129], and thus has the potential to mitigate the symptoms observed in RP.

Rho inhibitors: As described earlier, Rho-kinases have been involved in the propagation of cold-induced vasoconstriction by redistributing the α2C-adrenoreceptors from the Golgi apparatus to the cell membrane in response to the ROS released by the mitochondria upon stimulation by cold. Thus, Rho inhibitors, owing to their potential effects in the treatment of RP, have become significantly important. An example of a Rho inhibitor employed in vascular disorders is fasudil [130], which has also been observed to have benefits in pulmonary hypertension [131], thus opening a new door for investigating better treatment options for Raynaud’s.

Alternative approaches

The adverse side effects associated with pharmacologic therapies make patients adopt alternative approaches. Herbal drugs including *Ginkgo biloba* extracts such as seredine are of particular interest in the treatment of RP [132]. Essential rosemary oil applied topically has also been reported to have a vasodilatory and a warming effect [133]. The technique of acupuncture has also been introduced for managing RP and has been demonstrated to reduce the number of attacks and provide pain relief [134,135]. Auricular electroacupuncture has also been reported to mitigate the intensity and number of vasospastic episodes [136]. Low level laser therapy has also been observed to mitigate the severity and frequency of Raynaud’s attacks [137]. Improvements have also been reported with high-peak power laser treatment [138]. Surgical management of Raynaud’s has also been proposed. Thoracoscopic sympathectomy is indicated in patients with severe symptoms and is mainly to provide pain relief [108]. Adventitial stripping of digital arteries is another approach that has been employed. However, since results are anecdotal, the use of this approach is generally discouraged [139,140]. Percutaneous sympathetic block through mepivacaine and bupivacaine has also been demonstrated to be beneficial [141]. Vasodilation through transcutaneous nerve stimulation has also been employed in some cases [142]. In severe cases of RS, the use of spinal cord stimulators has also been seen [143]. As RP is a frequent occurrence in SSc, ustekinumab [144] and paquinimod [145] have been shown to provide benefit in relieving skin tightening associated with SSc. Besides these, general measures and topical treatments can also produce favorable results. Behavioral therapies, including biofeedback, have been investigated for their efficacy in the treatment of this phenomenon. However, the results of teaching temperature control techniques to patients have been less beneficial [146].

## Conclusions

RP is a commonly prevalent vasospastic disorder that can be an indication of a severe underlying pathology. Hence, it becomes imperative to thoroughly investigate the possibility of primary or secondary RP and to establish a clear distinction between the two through a detailed history, proper examination, thorough bloodwork, and imaging techniques. RP often goes unrecognized and unreported because milder vasospastic attacks are generally mislabeled as a typical response to severe cold. Owing to its complicated pathophysiology, a specific cause of RP is still a matter under debate, and future studies are warranted to uncover further aspects of its complex pathogenesis. The involvement of both central and local mechanisms further demands rigorous research. With occurrence of RP in parts other than digits, more investigations are needed to explore the pathomechanism of this condition. Moreover, as RP is aggravated by several factors, this further impedes the establishment of a solid pathology. In terms of treatment, vasodilators, besides calcium channel blockers, are deemed to be less effective in the treatment of digital artery occlusion and render adverse side effects. Exploration of new treatment strategies and drug approaches is desperately needed. Thus, both the cause and the treatment of RP stand in need of rigorous research so that a universal and gold-standard approach can be devised to relieve the symptoms associated with this condition.
